# Association of Motor and Cognitive Symptoms with Health-Related Quality of Life and Caregiver Burden in a German Cohort of Advanced Parkinson's Disease Patients

**DOI:** 10.1155/2020/5184084

**Published:** 2020-02-24

**Authors:** M. Klietz, T. Schnur, S. Drexel, F. Lange, A. Tulke, L. Rippena, L. Paracka, D. Dressler, G. U. Höglinger, F. Wegner

**Affiliations:** ^1^Department of Neurology, Hannover Medical School, Carl-Neuberg-Straße 1, 30625 Hannover, Germany; ^2^Behavioral Engineering Research Group, KU Leuven, Naamsestraat 69, 3000 Leuven, Belgium

## Abstract

Parkinson's disease (PD) is a chronic progressive movement disorder with severe reduction in patients' health-related quality of life (HR-QoL). Motor and cognitive symptoms are especially linked with decreased PD patients' HR-QoL. However, the relationship of these symptoms to caregiver burden is relatively unclear. Influence of the Montreal Cognitive Assessment scale (MoCA) as a cognitive screening tool and Movement Disorders Society Unified Parkinson's disease Rating Scale MDS-UPDRS symptoms in relation to patients' HR-QoL and caregivers` burden was analyzed. PD patients (*n* = 124) completed MDS-UPDRS, MoCA, and the PD questionnaire 8 (PDQ-8) as a measure of quality of life. Caregivers (*n* = 78) were assessed by the PD caregiver burden inventory (PDCB). PDQ-8 and PDCB scores were regressed on MDS-UPDRS subscales and MoCA subscores. PDQ-8 correlated with attention (*R*^2^ 0.1282; *p* < 0.001) and executive (*R*^2^ 0.0882; *p* 0.001) MoCA subscores and all parts of the MDS-UPDRS. PDCB correlated most strongly with MDS-UPDRS part III motor symptoms (*R*^2^ 0.2070; *p* < 0.001) and the MoCA attention subscore (*R*^2^ 0.1815; *p* < 0.001). While all facets of PD symptoms assessed by the MDS-UPDRS relate to PD patients' quality of life, motor symptoms are the most relevant factor for the prediction of caregiver burden. In addition, patients' attentional symptoms seem to affect not only them, but also their caregivers. These findings show the potential of a detailed analysis of MDS-UPDRS and MoCA performance in PD patients.

## 1. Introduction

Parkinson's disease (PD) is a common progressive neurodegenerative disease with a prevalence estimated from German health insurance data of up to 4,00,000 people in Germany [1]. Rigidity, tremor, and bradykinesia are the typical motor features of PD. Progression of PD leads to reduced quality of life and increasing burden for the caregiver [2–6].

After its revision in 2008, the MDS-UPDRS is the most important clinical score for rating of motor symptoms, nonmotor symptoms, and treatment complications in PD [7]. MDS-UPDRS symptom scores have been shown to relate to patients' health-related quality of life (HR-QoL) in international studies [6–8]. In contrast, the contribution of MDS-UPDRS symptoms to caregiver burden has only been investigated in smaller patient and caregiver samples [8, 9].

Another important factor known to correlate with poor HR-QoL is cognitive impairment, which is also suspected to correlate with increased caregiver burden [4, 10, 11]. PD patients whose symptoms frequently include cognitive impairments may progress to dementia during advanced stages of the disease [12, 13]. One of the most established tests for cognitive assessment in PD is the Montreal Cognitive Assessment (MoCA) [14, 15]. Recently, subdomains of the test have been defined to enable a more specific examination of cognition [16]. The correlation of these MoCA subdomains to HR-QoL and caregiver burden has not yet been studied in PD patients. Furthermore, depression has huge influence on patients' and caregivers' HR-QoL [4, 17]. One of the most established tools for assessment of depressed mood is the Beck Depression Inventory (BDI) [18]. Besides, depressive symptoms can be evaluated using the MDS-UPDRS part I (Nonmotor Aspects of Experiences of Daily Living) [7].

This study aims to investigate the contribution of distinct MDS-UPDRS parts and MoCA subdomains to patients' HR-QoL and caregiver burden in a German monocentric cross-sectional sample of PD patients and their caregivers. Based on prior research, we expect to find positive correlations of MDS-UPDRS and MoCA scores with HR-QoL and caregiver burden.

## 2. Methods

### 2.1. Participants

We obtained approval from the local Ethics Committee of Hannover Medical School (no. 3178-2016, Amendment in 2018). Our sample included 124 PD patients and 78 caregivers of PD patients, all of whom gave their written informed consent. Inclusion criteria for PD patients were defined as neurologically determined Parkinson's disease with a duration of at least one year. All included patients met the MDS-diagnosis criteria for PD [19] and none of the PD patients was considered as atypical Parkinsonism. In all PD patients, a movement disorder specialist confirmed the diagnosis of PD. Patients suffering from known atypical Parkinsonism were excluded from this study. Education of the PD patient was assessed on ordinal level as low, middle, or high (as also conducted in [20]). A primary caregiver was defined as a person that serves as the primary care provider. Only the primary caregiver (e.g., mostly spouses) of the PD patients was included. In the case of multiple caregivers, only the caregiver spending the most time with the patient and handling most of the care issues was included for the study. Professional caregivers were excluded from our study.

### 2.2. Measures

PD patients were examined by using MDS-UPDRS [7] (assessment of PD symptoms, in the best medical “on state”) and MoCA (cognitive screening test: ranging from 0 to 30 points, 30 to 26 points were considered as normal cognitive function, 25 to 21 points as mild cognitive impairment, and below 21 points as suspicious for dementia, performed in the clinical “on state”) [3, 4]. In order to asses HR.QoL, patients were provided with a copy of Parkinson's disease quality of life questionnaire 8 (PDQ-8) [21]. This scale assesses patients' functioning and well being across eight domains. Patients had to answer 8 questions on a 5-point Linkert scale ranging from 0 to 4. Higher point values indicate more restrictions in HR-QoL of the patient. All 8 items were added together (0–32 points possible in total) suggesting the percentage of HR-QoL restrictions after recalculation. Higher percent scores (range from 0–100) indicate worse HR-QoL restrictions of the PD patient. Caregivers were asked to assist patients who show possible cognitive impairment in completing the PDQ-8 to ensure correct results and avoid anosognosia (as also conducted in [4, 17]). During the study design, we discussed taking the PDQ-39 or the PDQ-8 and decided to take the short form because of the almost identical psychometrics and increased compliance of the participants due to reduction of items [21].

Caregivers were asked to complete a newly developed and validated German version of Parkinson's disease caregiver burden questionnaire (PDCB) [15, 16]. In brief, the PDCB includes 20 items on a five-point Likert scale (0 to 4). Participants can reach a maximum of 80 points on this part of the questionnaire. In addition, participants had to rate their global burden as a caregiver on a scale from 0 to 100 in the second part of the questionnaire. Finally, points from the first part and 20% of the global burden were summed up for the total caregiver burden score. A maximal score of 100 can be reached indicating highest possible burden.

Caregiver and patient depression was assessed by Beck´s depression inventory [18] (BDI, depressive symptoms ranging from 0–63 points: 0–13: no depression, 14–19: mild depression, 20–28: moderate depression, and 29–63: severe depression). Furthermore, HR-QoL of the caregivers was measured by the short 36 health survey (SF-36), which measures health-related quality of life across eight dimensions [22]. Subscale scores were percentage-transformed so that a score of 0 would indicate maximum impairment and a score of 100 would indicate the absence of any reported impairment. Since all subscale scores were highly correlated, we calculated an average score across the eight scales [17, 23].

Caregivers and patients were also asked to provide general information about their background and demographics (e.g., patients' disease duration as well as the daily amount of time caregivers dedicated to giving care to the patient).

### 2.3. Analyses

Linear regression analyses were used to examine potential predictors of PDQ-8 and PDCB scores. To correct for multiple testing, the level of significance was set to *α* = 0.05/*n* (with *n* being the number of analyzed predictors). To test whether any predictors explained unique variance in our outcomes, we included significant predictors in a multiple regression model for patients' HR-QoL and caregiver burden. Analyses were carried out using SPSS 25.0 (IBM, Armonk, NY). The biometric institute of Hannover Medical School, Hannover, Germany, counseled these statistical analyses.

## 3. Results

### 3.1. Patient and Caregiver Characteristics

Demographic and clinical characteristics of PD patients and caregivers are displayed in Table 1, which reveals substantial variation in both outcome variables. Similarly, patients varied considerably with regard to PD-related impairment (as indicated by MDS-UPDRS scores) and cognitive status (as indicated by MoCA scores). Twenty-two patients (21%) presented with normal cognition indicated by a MoCA score of 26 or higher (Table 2). Cognitive impairment was found in 37.1% (*n* = 46) of the patients, and 41.9% (*n* = 53) of the patients scored a MoCA result suggestive of dementia (<21) (Table 2). Regression analyses are thus unlikely to be affected by variance restriction.

### 3.2. Factors Influencing Quality of Life in PD Patients

To assess whether variance in patients' HR-QoL can be explained by the severity of their PD symptoms or cognitive impairment, PDQ-8 scores were regressed on MDS-UPDRS and MoCA scores. After correction for multiple comparisons, all parts of the MDS-UPDRS were found to correlate significantly with PDQ-8 scores. Similarly, PDSI scores were significantly related to the attention index score and executive index score of the MoCA. Better performance in these domains and lower MDS-UPDRS scores predicted better HR-QoL. Further analyses revealed that patients' HR-QoL also decreased with increasing BDI scores and disease duration. All data from these linear regression analyses are displayed in Table 3.

Furthermore, we tested against cocorrelation and added all significant variables in a multiple regression analysis. After 4 rounds of backward-selection, we found MDS-UPDRS II and BDI significantly correlating with HR-QoL of the PD patients. MDS-UPDRS II (*R*^2^ 0.2007; beta 0.389 (95%CI 0.170–0.727); *p* 0.002) explains 15.1% of variance in the analysis, and BDI (*R*^2^ 0.0661; beta 0.247 (95%CI 0.013–0.501); *p* 0.040) explains 6% of variance in the paradigm.

### 3.3. Factors Influencing Caregiver Burden in PD Caregivers

Factors we evaluated in relation to caregiver burden are displayed in Table 3. In our study, caregiver burden measured by the PDCB as PD specific-questionnaire correlated significantly after correction for multiple testing with MDS-UPDRS part III, PDQ-8, and caregivers' BDI (Table 4^*∗∗*^). Furthermore, we tested against cocorrelation and added all significant variables correlating with caregiver burden in a multiple regression analysis. After 6 rounds of backward-selection, we found only PDQ-8 (*R*^2^ 0.0876; beta 0.294 (95%CI −0.091–0.696); *p* 0.064) and MDS-UPDRS part III (*R*^2^ 0.1069; beta 0.272 (95%CI −0.148–0.688); *p* 0.086) exposing a tendency to correlate with caregiver burden.

## 4. Discussion

In this study, we evaluated the contribution of MDS-UPDRS and MoCA subscores to HR-QoL of PD patients and caregivers' burden measured by the disease-specific, validated PDCB [17, 24]. We found all parts of the MDS-UPDRS as well as the attention and executive index of the MoCA correlating significantly with PDQ-8 as measure of patients' HR-QoL. Concerning caregiver burden, we found significant correlations of the PDCB with part III of the MDS-UPDRS and the MoCA attention index.

### 4.1. Association of MDS-UPDRS, MoCA Subscales, and Patients' Quality of Life

Patients with PD suffer from a variety of clinical symptoms, which include motor symptoms like rigidity, tremor, bradykinesia, and postural instability [25]. Furthermore, PD patients suffer from a large number of nonmotor symptoms, which impact HR-QoL in an extensive manner [26]. The revised version of the MDS-UPDRS is the gold standard for assessment of PD symptoms [7]. It includes nonmotor symptoms in part I of the scale, impact of motor symptoms on daily living in part II, actual motor-deficits at time point of the consultation in part III, and motor complications in part IV. Several studies recently reported a good correlation of MDS-UPDRS total score and the subparts with HR-QoL [27–29]. In our sample, we can confirm these results showing significant correlations of all parts of the MDS-UPDRS and HR-QoL measured by the PDQ-8. As expected, we found the strongest correlations for nonmotor-impairment and motor-impairment in daily living of the patient represented in part I and II. These data are in line with Rodriguez-Blázquez et al. showing MDS-UPDRS II to have the greatest impact on HR-QoL [27]. Motor-impairments in daily living collected throughout the course of one week might be a more robust indicator for functional impairment than a clinical examination and even correlate with metabolic changes in the brain of early PD patients [30]. Besides, motor-examination is influenced by a large variety of important factors like the medical status of the patient. In our study, we examined all patients in the best medical on state; therefore, the extent of motor-impairment might have been underestimated in relation to the off state [4, 30].

Cognitive impairments are frequent in PD and may already occur in early stages of the disease [13]. However, the conversion of cognitive decline to dementia mainly develops in advanced or late stages of PD [12]. In our cohort, we report data from predominantly long-term PD patients with an average disease duration of 14.1 years. This fact may account for the large proportion of cognitively impaired patients in our sample, which is in line with data from other cohorts of advanced PD patients [4, 31, 32]. Cognitive impairments represent only one item in the MDS-UPDRS part I and seem underrepresented concerning the dramatic impact on patients' quality of life [32]. By using recently published subdomains of the MoCA, we were able to zoom in on those cognitive functions that account for the link between cognitive impairment and HR-QoL in PD patients. We found deficits in the executive and the attentional domain to be substantially related to patients' quality of life. Executive and attentional functional deficits are well described in PD patients [33–37]. Nevertheless, a recent study reports that executive dysfunction might be ameliorated by dopamine, whereas global cognitive deficits were not improved [38]. Since we examined all patients in the on state, executive function might be even underestimated in our cohort. Future studies might want to focus more on executive and attention training in these patients to finally increase their cognitive abilities and HR-QoL [39].

### 4.2. Association of MDS-UPDRS, MoCA, and Caregiver Burden

Zarrit et al. defined caregiver burden as a multifactorial construct summarizing the adverse impacts that giving care for someone else have on the caregiver [40]. Recently, Vatter et al. defined these dimensions as social and psychosocial constrain, personal strain, interference with personal life, concerns about the future, and guilt [41]. However, patient factors and caregiver factors contribute to the absolute perceived burden of the caregiver [42]. Importantly, caregivers are at risk of burn out as their burden exacerbates which may lead to institutionalization of the patient [43, 44]. Especially in PD, caregivers face a long phase of progressive disability of the patients with decline in motor and also cognitive abilities.

We were interested in assessing the impact of specific MDS-UPDRS parts and patient cognition on caregivers' burden measured by the disease-specific and validated PDCB [10, 17, 45]. Rodriguez-Violantes et al. reported MDS-UPDRS part II as most robustly correlating with caregiver burden measured by the Zarit Burden Inventory [9]. In contrast, in our study, part III of the MDS-UPDRS emerged as the only significant correlate of caregiver burden after correcting for multiple comparisons. Part III accounted for more than twice as much variance in caregiver burden than part II. This finding is at odds with data presented by Grün et al. who found only a weak association of the MDS-UPDRS III with caregiver burden in a smaller study in Luxemburg [42]. In a cohort of advanced PD and patients with atypical Parkinsonism, Schmotz et al. did not find a correlation of motor symptoms with caregiver burden in both groups [46]. Data from various groups have shown that caregiver burden is even more related to nonmotor symptoms of the patient than to motor symptoms [46–50]. However, most of these studies were performed by using a non-PD-specific questionnaire for caregiver burden. It remains speculative if the PDCB is too much focused on burden induced by motor symptoms and less able to detect nonmotor symptom-related caregiver burden.

In our study, we also found a significant correlation of the PDCB score with the attention index of the MoCA. These data are supported by a study from the UK finding attentional deficits to be most strongly associated with caregiver burden [51]. Hence, it appears that impaired attentional functioning in patients with PD does not only affect patients (as indicated by its contribution to quality-of-life impairment) but also their caregivers.

Auxiliary analyses further revealed caregiver burden to be significantly related to patients' quality of life in our study. This has also been demonstrated in the German validation study of the PDCB for the PDQ-39 [17]. Moreover, depressive symptoms of the caregiver correlated significantly with caregiver burden. This association is well described in the context of caregiver burden and an important factor with regard to caregiver burnout [43]. Caregiving hours per day are also an important correlate of perceived burden [17, 44, 49]. By connecting the individual findings and taking all collected evidence into account, this study showed the important association of motor and nonmotor symptoms with caregiver burden and further propagates the PDCB as a disease specific and valid assessment tool for caregiver burden in PD.

### 4.3. Limitations

We report data from a monocentric cross-sectional observational study. Despite the good sample size (*n* = 124) for a monocentric study, we were not able to reach patient numbers of other MDS-coordinated analyses. Since we were aware of the pronounced cognitive deficits in the study sample, we usually made a cognitive pretest and integrated the caregiver in the assessment of the patient if necessary. However, we could not exclude some anosognosia in the patient sample and, therefore, a possible underestimation of the patient impairment. We used the MoCA as cognitive screening tool for cognitive impairments and analyzed the subdistribution of these deficits in PD patients. Nevertheless, a cognitive screening tool cannot replace an extensive cognitive testing and is not sufficient to raise the diagnosis of PD-related dementia. The patient's educational level was only assessed for the MoCA screening, and in an ordinal way, we did not correct the analysis for years of education of the individual patient. Another restriction is that we only report cross-sectional data from one time-point, while longitudinal data would be desirable to ensure that motor and cognitive symptoms precede the decreases in HR-QoL and evaluation of caregiver burden described in this study. Since patients were free to decide to participate in our study, we cannot exclude a selection bias including more motivated patients and their caregivers. While we did not evaluate patients' medication and comorbidities in our study, we only included patients without severe comorbidities [52].

## 5. Conclusion

MDS-UPDRS is the gold standard measure for the assessment of PD symptoms and shows a robust correlation with patient HR-QoL. It provides assessment of nonmotor symptoms, motor symptoms, and motor complications. However, while having an extraordinary importance for patients' HR-QoL and caregiver burden, cognitive symptoms are underrepresented in the MDS-UPDRS, which indicates that the use of an additional cognitive screening test would be reasonable. The MoCA is excellently validated for PD, and recent subscores provide a clinically relevant possibility to further differentiate cognitive impairments. We show that executive and attentional impairments in particular contribute to PD patients' HR-QoL. Caregiver burden measured by the PDCB showed the most robust correlation with the MDS-UPDRS part III in the best medical on, confirming the disease specificity of the assessment tool. In addition, caregiver burden seems to relate patients' attentional deficits. Further studies are required to explore possible means to mitigate the effect of PD symptoms on HR-QoL and caregiver burden.

## Figures and Tables

**Table 1:** Patient (*n* = 124, 61 females) and caregiver (*n* = 78, 42 females) characteristics.

	Mean ± SD	Min	Max
PD patients			
Age (years)	72.5 ± 9.1	40	89
Education	Low 1.8%		
	Middle 61.4%		
	High 36.8%		
Disease duration (years)	14.1 ± 7.6	1	37
Hoehn and Yahr stage	3.5 ± 1.1	1	5
MDS-UPDRS total	115.0 ± 47.7	12	210
MDS-UPDRS part I	17.4 ± 8.4	1	39
MDS-UPDRS part II	25.2 ± 11.9	1	48
MDS-UPDRS part III	48.1 ± 21.8	3	96
MDS-UPDRS part IV	7.2 ± 5.6	0	20
PDQ-8	40.4% ± 18.0%	0%	81.3%
MoCA	20.4 ± 6.7	0	30
MoCA-EIS	5.6 ± 5.2	0	15
MoCA-VIS	3.8 ± 1.2	0	5
MoCA-AIS	12.0 ± 4.8	0	18
MoCA-OIS	5.3 ± 1.5	0	9
MoCA-MIS	5.8 ± 5.2	0	15
MoCA-LIS	4.6 ± 1.4	0	6
BDI	11.3 ± 6.9	0	31
Barthel index	70.6 ± 26.5	10	100

Caregivers			
Age (years)	64.8 ± 11.0	19	84
Caregiving hours per day	5.9 ± 7.0	0	24
PDCB	36.5 ± 27.1	0	100
BDI	9.3 ± 6.3	0	26
SF-36 total	67.4 ± 19.0	21.9	96.3

BDI, Beck Depression Inventory; PDCB, Parkinson's disease caregiver burden inventory; MDS-UPDRS, Movement Disorders Society-unified Parkinson's disease rating scale; MoCA, Montreal cognitive assessment scale; EIS, executive index score; VIS, visual index score; AIS, attention index score; OIS, orientation index score; MIS, memory index score; LIS, language index score; PD, Parkinson's disease; PDQ-8, Parkinson's disease questionnaire 8; SF-36, short form 36 health survey; SD, standard deviation.

**Table 2:** Characteristics and distribution of MoCA subscores in PD patients with normal cognition, cognitive impairments, and patients suspicious for dementia.

	Normal cognition *n* = 22	Impaired *n* = 46	Dementia *n* = 53
MoCA	27.9 ± 1.3	23.6 ± 1.5	14.5 ± 5.8
MoCA-EIS	70.7% ± 4.7%	60.0% ± 9.3%	35.3% ± 18.6%
MoCA-VIS	90.0% ± 10.0%	86.0% ± 1.4%	62.0% ± 26.0%
MoCA-AIS	88.9% ± 13.8%	74.4% ± 16.1%	51.1% ± 28.3%
MoCA-OIS	67.8% ± 6.7%	65.6% ± 3.3%	48.9% ± 20.0%
MoCA-MIS	80.7% ± 18.7%	47.3% ± 27.3%	13.3% ± 23.3%
MoCA-LIS	95% ± 10%	83.3% ± 13.3%	61.7% ± 26.6%
PDQ-8	30.5% ± 17.1%	38.3% ± 20.0	44.8% ± 14.1%
BDI	10.0 ± 4.5	9.5 ± 7.1	12.5 ± 6.2
HaY stage	2.6 ± 1.1	3.2 ± 1.0	4.0 ± 0.8
Disease duration	11.1 ± 6.4	13.2 ± 7.1	16.2 ± 7.8
Age	64.2 ± 10.7	71.5 ± 7.4	77.4 ± 6.0
PDCB	31.6 ± 25.4	27.0 ± 25.1	49.0 ± 25.3

Participating patients were grouped in 3 categories: normal cognition (MoCA 30-26 points), cognitively impaired (MoCA 25-21 points), and dementia (MoCA below 21 points). MoCA subscores were displayed in percentage for better comparability between different subscores. Since we wanted to avoid loss of information in group effects, we did not calculate an ANOVA analysis on these data. Missing data: 3 patients. BDI, Beck Depression Inventory; HaY, Hoehn and Yahr; MoCA, Montreal cognitive assessment scale; EIS, executive index score; VIS, visual index score; AIS, attention index score; OIS, orientation index score; MIS, memory index score; LIS, language index score; PDQ-8, Parkinson's disease questionnaire 8; PDCB Parkinson's disease caregiver burden questionnaire.

**Table 3:** Linear regression analysis of factors influencing patient health-related quality of life (PDQ-8), *n* = 124.

Variables	*R* ^2^	Beta	*p*
Age	0.0262	0.162	0.074
Disease duration	0.1005	0.315	**<0.001** ^*∗∗*^
MDS-UPDRS total	0.2209	0.472	**<0.001** ^*∗∗*^
MDS-UPDRS part I	0.1910	0.478	**<0.001** ^*∗∗*^
MDS-UPDRS part II	0.1892	0.440	**<0.001** ^*∗∗*^
MDS-UPDRS part III	0.0999	0.319	**<0.001** ^*∗∗*^
MDS-UPDRS part IV	0.0936	0.306	**0.001** ^*∗∗*^
MoCA	0.0566	−0.240	0.008
MoCA-EIS	0.0882	−0.296	**0.001** ^*∗∗*^
MoCA-VIS	0.0784	−0.247	0.006^*∗*^
MoCA-AIS	0.1282	−0.260	**<0.001** ^*∗∗*^
MoCA-OIS	0.0562	−0.220	0.016^*∗*^
MoCA-MIS	0.0119	−0.110	0.231
MoCA-LIS	0.0106	−0.101	0.270
BDI	0.2089	0.445	**<0.001** ^*∗∗*^

Linear regression analysis of factors contributing to patients' health-related quality of life. *p* value adjustment for multiple comparisons was 0.05/15. ^*∗*^Significant correlations at *p* < 0.05; ^*∗∗*^Significance level at *p* < 0.003 printed in bold. BDI, Beck Depression Inventory; MDS-UPDRS, Movement Disorders Society unified Parkinson's disease rating scale; MoCA, Montreal cognitive assessment scale; EIS, executive index score; VIS, visual index score; AIS, attention index score; OIS, orientation index score; MIS, memory index score; LIS, language index score; PDQ-8, Parkinson's disease questionnaire 8.

**Table 4:** Linear regression analysis of factors influencing caregiver burden (PDCB), *n* = 78.

Variables	*R* ^2^	Beta	*p*
PD patient BDI	0.0292	0.165	0.229
MDS-UPDRS part I	0.0812	0.287	0.012^*∗*^
MDS-UPDRS part II	0.0818	0.282	0.014^*∗*^
MDS-UPDRS part III	0.2070	0.433	**<0.001** ^*∗∗*^
MDS-UPDRS part IV	0.0467	0.219	0.058
MoCA	0.0625	−0.254	0.029^*∗*^
MoCA-EIS	0.0524	−0.222	0.057
MoCA-VIS	0.0480	−0.176	0.134
MoCA-AIS	0.1815	−0.417	**<0.001** ^*∗∗*^
MoCA-OIS	0.0110	−0.098	0.405
MoCA-MIS	0.0445	−0.212	0.070
MoCA-LIS	0.0818	−0.284	0.014^*∗*^
PDQ-8	0.1840	0.436	**<0.001** ^*∗∗*^
CG-SF-36 total	0.0243	−0.157	0.193
CG-h/d	0.1069	0.305	0.037^*∗*^
CG-BDI	0.1560	0.402	**<0.001** ^*∗∗*^

*p* value adjustment for multiple comparisons was 0.05/16. ^*∗*^Significant correlations at *p* < 0.05; ^*∗∗*^significance level at *p* < 0.003. BDI, Beck Depression Inventory; CG, caregiver; MDS-UPDRS, Movement Disorders Society-unified Parkinson's disease rating scale; MoCA, Montreal cognitive assessment scale; EIS, executive index score; VIS, visual index score; AIS, attention index score; OIS, orientation index score; MIS, memory index score; LIS, language index score; PDCB Parkinson's disease caregiver burden inventory; PDQ-8, Parkinson's disease questionnaire 8; and SF-36, short form 36 health survey.

## Data Availability

Data are available on reasonable request from the corresponding author.
